# Quantification of codon selection for comparative bacterial genomics

**DOI:** 10.1186/1471-2164-12-374

**Published:** 2011-07-25

**Authors:** Adam C Retchless, Jeffrey G Lawrence

**Affiliations:** 1 Department of Biological Sciences, University of Pittsburgh, Pittsburgh, PA 15260, USA; 2 Department of Environmental Science, Policy and Management, University of California, Berkeley, CA 94720, USA

## Abstract

**Background:**

Statistics measuring codon selection seek to compare genes by their sensitivity to selection for translational efficiency, but existing statistics lack a model for testing the significance of differences between genes. Here, we introduce a new statistic for measuring codon selection, the Adaptive Codon Enrichment (ACE).

**Results:**

This statistic represents codon usage bias in terms of a probabilistic distribution, quantifying the extent that preferred codons are over-represented in the gene of interest relative to the mean and variance that would result from stochastic sampling of codons. Expected codon frequencies are derived from the observed codon usage frequencies of a broad set of genes, such that they are likely to reflect nonselective, genome wide influences on codon usage (*e.g*. mutational biases). The relative adaptiveness of synonymous codons is deduced from the frequency of codon usage in a pre-selected set of genes relative to the expected frequency. The ACE can predict both transcript abundance during rapid growth and the rate of synonymous substitutions, with accuracy comparable to or greater than existing metrics. We further examine how the composition of reference gene sets affects the accuracy of the statistic, and suggest methods for selecting appropriate reference sets for any genome, including bacteriophages. Finally, we demonstrate that the ACE may naturally be extended to quantify the genome-wide influence of codon selection in a manner that is sensitive to a large fraction of codons in the genome. This reveals substantial variation among genomes, correlated with the tRNA gene number, even among groups of bacteria where previously proposed whole-genome measures show little variation.

**Conclusions:**

The statistical framework of the ACE allows rigorous comparison of the level of codon selection acting on genes, both within a genome and between genomes.

## Background

It has long been recognized that protein-coding sequences show nonrandom, organism-specific patterns of codon usage [1]. Codon usage bias is most pronounced in highly expressed genes [2], where codon preferences are associated with the tRNA abundance within the cytoplasm [3]. Measurement of codon selection is of interest because the extent to which different genes use the preferred codons is predictive of their expression levels and rates of evolutionary change [4-6], and thus their relative importance (in terms of transcript abundance and degree of conservation) to the organism. Comparative studies of codon selection have provided insight into the population structure and lifestyle of organisms [7-14]. Numerous statistics have been devised to measure variation in codon selection among Open Reading Frames (ORFs) within a genome, yet none fully account for the evolutionary dynamics that shape codon usage bias, including compositional differences among genes and genomes. The simplest metrics evaluate how much codon usage frequencies of a gene deviate from expected frequencies. These methods, such as the Effective Numbers of Codons (ENC) and the ENC' [15,16], incorporate no information about the fitness differences among synonymous codons. This limitation has been addressed by Karlin and Mrazek [7] and Supek and Vlahovicek [17,18], whose algorithms simultaneously compare each gene's codon usage both to genome-wide codon frequencies (representing mutational tendencies) and to codon frequencies in a defined set of genes believed to experience strong codon selection. However, this design has been criticized for failing to assign the most extreme values to the genes with the most extreme biases in terms of preferred or non-preferred codons [19]. This artifact results from the maximum possible value being assigned to genes with codon composition identical to pre-selected set of "optimized" genes, even though other genes may show more extreme enrichment of the optimal codons.

This irregularity is absent from statistics that are proportional to the frequency at which preferred codons occur within an ORF. At their simplest, these statistics summarize the optimal codon frequency for each amino acid (*e.g*. F_op _[3] and CBI [20]) while more complicated methods construct a scoring table for all codons, quantifying the relative importance of non-optimal codons and weighting the statistic so that it is influenced more by those amino acids for which the synonymous codons have a greater perceived fitness difference (*e.g*. CAI [21], tAI [22], GCB [23]). One method for normalizing across amino acids is to compare the score of the observed codons against the maximum possible score for an ORF with the same amino acid composition (*e.g*. CAI, tAI), producing a uniform maximum score for all ORFs regardless of amino acid composition. However, this does not account for the fact that the probability of observing the optimal codon will vary according to amino acid composition, and the values assigned to non-optimal codons can vary greatly among amino acids [24]. Despite the power of these methods for detecting codon selection, none of them quantify the stochastic variation that is expected to arise from mutation-selection balance, which is the primary explanation for the occurrence of non-optimal codons [25,26]. The selection-mutation-drift theory of synonymous codon usage describes an equilibrium condition where preferred and non-preferred codons occur in proportions determined by mutational biases, selection, and effective population size. Recent studies have calculated the parameters of this model explicitly [8,24,27], but only include codons for two-fold degenerate amino acids, limiting the information available to make inferences about individual genes. To date, no analytical method accounts for the variation in the codon usage statistic that arises from the stochastic nature of the selection-mutation-drift model.

Here, we expand upon the scoring-table class of methods by introducing a new statistic that incorporates a table of expected codon frequencies, which amounts to a null hypothesis for codon usage. We present a stochastic model of codon usage, thereby allowing ORFs to be evaluated in terms of their deviation from an expected codon composition. This not only allows us to measure the impact of selection against the background of genome-wide biases, but to normalize the values assigned to non-preferred codons of different amino acids so that amino acid composition does not affect the score under the null model. We also examine different algorithms for systematically assessing codon frequencies - either in the presence or absence of selection - using only the genome sequence of the organism being examined. By deriving the expected distributions of the statistic under a null hypothesis about codon frequencies, our statistical framework provides a means to compare the strength of codon selection within and between genomes.

## Results

Below, we describe a statistic for summarizing the codon usage of an ORF. The raw statistic is the sum of values assigned to each of the codons in the sequence and may be normalized according to its expected distribution. Normalized scores for individual genes can be combined to summarize the magnitude of codon selection operating on the entire genome. We compare our measure to previously described codon usage statistics, both conceptually and empirically.

### Relative Adaptiveness of Synonymous Codons

To quantify enrichment of a codon among genes experiencing codon selection, we define a score (δ) for each codon *cdn *as,(1)

where *cdn_ij _*is the *j*th codon of the *i*th amino acid and *f*(*cdn_ij_*) is the expected frequency of that codon among its synonyms in genes that have (*f_o_*) or have not (*f_n_*) been optimized by codon selection. Use of the logarithm enables us to express the codon optimization of a gene or set of genes as the sum of the individual scores of the codons comprising the gene, generating the Summed Codon Bias (*SCB*). To facilitate examination of the stochastic properties of the *SCB*, it is calculated as the sum of the composite scores for each amino acid (α), which are determined from the scores of their constituent codons as,(2)(3)

where *C_ij _*is the count of that codon within the gene being analyzed and *N_i _*is the number of synonyms for its encoded residue. Merkl proposed a similar statistic, the GCB (where his constituent CB is equal to our δ) arguing that this form of statistic is optimal for distinguishing between two populations[23]. Here, we use the sum because it has convenient properties in a stochastic model, described below, which we will use to normalize this continuous statistic. Notably, the *SCB *expresses codon usage bias as a function of the difference (δ) between unselected (*f_n_*) and selected (*f_o_*) codon frequencies, rather than as a distance from them, thus avoiding shortcomings of other metrics [19].

The *SCB *is related to other scoring-table statistics by different normalization routines. Merkl's GCB [23] is the length-normalized form of the *SCB*. The logarithm of the CAI [21] can be derived from the *SCB *by calculating δ*_ij _*with a non-optimized table (*f_n_*) showing no bias among synonymous codons, then calculating the difference between *SCB *and the maximum possible value given its amino acid composition, and finally dividing by the number of codons in the ORF, ignoring methionine and tryptophan.

Crucially, scoring tables created from δ*_ij _*reveal which codons increase in frequency among the most optimized proteins, and to what degree. This is different from the Relative Synonymous Codon Usage values that are used to calculate the CAI [21], which reflect simply the abundance of codons in optimized genes without reference to their abundance in non-optimized genes. Codons with greatest abundance in optimized genes may not have experienced the strongest selection for enrichment and, in the worst cases, may actually be disfavored. This adjustment to the estimate of codon adaptiveness should have the greatest effect in genomes where nucleotide composition shows the greatest deviation from equal usage.

To examine the effect of this difference between *SCB *and CAI, we evaluated multiple genomes by constructing *f*_o _from the synonymous codon frequencies of a set of 40 protein-coding genes whose products comprise the ribosome and other parts of the translation apparatus [8] (henceforth, "Translation40", see Methods) and constructing *f_n _*from all ORFs in the genome. Accounting for the biases in *f_n _*creates substantial changes in δ relative to the values obtained otherwise (Table 1), even changing estimates of which codon is most preferred. In *Pseudomonas putida *(67% GC), for four amino acids, the synonymous codons that are enriched among ribosomal proteins and translation elongation factors are not the same as the synonymous codons that are most abundant among those proteins. These effects are also observed in genomes with less bias in nucleotide composition, such as *Bacillus subtilis *(44% GC) and *Escherichia coli *(51% GC), each of which had one amino acid where the most enriched codon is not the most abundant codon.

**Table 1:** Normalized Synonymous Codon Usage as a function of alternative codon scoring tables.

		*Escherichia coli *MG1865			*Bacillus subtilis *168			*Pseudomonas putida *KT2440		
**Residue**	**Codon**	***f_n_*^1^**	***f_o_*^2^**	***δ *^3^**	***f_n_***	***f_o_***	***δ*^3^**	***f_n_***	***f_o_***	***δ***

Lys	AAG	0.303	0.380	1.000	0.427	0.189	0.441	1.000	**1.000**	0.634
Lys	AAA	1.000	**1.000**^**4**^	0.800	1.000	1.000	1.000	0.385	0.607	1.000
										
Pro	CCG	1.000	1.000	1.000	1.000	0.279*	0.127	1.000	**1.000**	0.409
Pro	CCA	0.358	0.183^5^	0.511	0.439	0.962	1.000	0.2817	0.689	1.000
Pro	CCT	0.295	0.206	0.697	0.659	**1.000**	0.693	0.2374	0.557	0.959
Pro	CCC	0.231	0.017	0.074	0.206	0.039	0.086	0.4627	0.151	0.134
										
Thr	ACG	0.613	0.082	0.050	0.652	0.233*	0.140	0.264	0.046	0.078
Thr	ACA	0.290	0.094	0.121	1.000	0.606*	0.238	0.104	0.054	0.232
Thr	ACT	0.374	1.000	1.000	0.392	1.000	1.000	0.137	0.307	1.000
Thr	ACC	1.000	0.924*^6^	0.346	0.386	0.026	0.026	1.000	**1.000**	0.448
										
Val	GTG	1.000	0.229*	0.160	0.906	0.168	0.185	1.000	0.646*	0.117
Val	GTA	0.415	0.545	0.916	0.695	0.629	0.904	0.201	0.399	0.361
Val	GTT	0.698	1.000	1.000	1.000	1.000	1.000	0.181	1.000	1.000
Val	GTC	0.587	0.139	0.166	0.904	0.157	0.174	0.572	0.798*	0.253

1. The *f_n _*table was constructed using all of the genes in the specified genome; NSCU values are the frequency of each codon normalized to the largest value within each synonymous codon group

2. The *f_o _*table was constructed using the Translation40 genes [8].

3. The normalized δ values were calculated as the *f_o_*/*f_n _*ratio, thus correcting *f_o _*values to the codon composition of the genome as a whole.

4. Bolded underlines indicate that the uncorrected table significantly underestimates selection against this codon and incorrectly denotes it as the preferred codon.

5. Single underlines indicate that the uncorrected table significantly overestimates selection against this codon.

6. Asterisks indicate that the uncorrected table significantly underestimates selection against this codon.

### Normalization to a theoretical distribution

Rigorous interpretation of any codon bias statistic depends upon knowledge of its distribution given expected synonymous codon usage frequencies. Issues as simple as discerning if one ORF is more enriched for optimal codons than another cannot be resolved unless we know the values that are expected to arise from ORFs that vary in amino acid composition but not synonymous codon frequencies. Likewise, unless the variance of the summary statistic is known, variation between genes cannot be inferred to result from differences in the strength of selection between those genes rather than being due to the stochastic nature of mutation and drift.

The expected codon frequencies will depend upon the null hypothesis being tested. If the null hypothesis is that an ORF has not been shaped by selection for optimal codons, then the table of expected codon frequencies for each amino acid is equivalent to *f_n_*, above. For now, we will use genome-wide codon composition as estimates of *f_n_*, although we will refine this estimate below. To estimate the distribution of the *SCB *expected for a given ORF, we first estimate the sampling distribution of the composite score for each amino acid (α). The expected score of each amino acid is the count (*C*) of that amino acid, multiplied by the weighted average of the scores of each of its codons (δ), so that(4)

and(5)

where *P_ij_*, the probability of observing that codon at random, is equivalent to *f_n_*(*cdn_ij_*). In our null model, the identity of the codon at each site is independent of those at other sites, meaning that the variance of the *SCB *is the sum of the variance for each site, so that(6)

and(7)

Being the sum of many independent random variables, the *SCB *has an approximately normal distribution according to the Central Limit Theorem [28]. Many statistical tests assume a normal distribution, so we will describe a statistic derived from that distribution. The Adaptive Codon Enrichment (ACE) is the difference between the observed *SCB *and the expected *SCB *for an ORF:(8)

This may be normalized in two ways. First, it may be presented as a standard deviation score or Z-value as,(9)

This statistic can be used in a Z-test to evaluate the probability that the codon composition of a gene differs significantly from that predicted from mutational bias alone. Alternatively, the ACE may be unit normalized so that it reflects the deviation averaged per codon in the coding sequence as,(10)

Because amino acids differ in their sensitivity to codon selection, they each contribute different amounts of variance to the final score, so normalization takes into account the variance contributed by each amino acid rather than simply dividing by the length of the encoded protein. The equation for ACE*_u _*is equivalent to the average of the Z-value for each individual codon. Notably, the ACE is indifferent to the inclusion or exclusion of methionine and tryptophan codons because, having only single codons, they influence the observed and expected values identically and thus contribute no variability. This is in contrast to statistics that are sensitive to the frequency with which the most preferred codon occurs, such as the CAI, where methionine and tryptophan are explicitly ignored [21].

To validate that ACE statistics can be treated as random normal variables, we used Monte Carlo simulations to examine the properties of genes for which the *SCB *fit this assumption. Distributions were constructed from 2000 Monte Carlo samples for each ORF of *E. coli *and *P. putida*, using the expected codon distribution of the respective genome. The predicted mean and variance were universally accurate, while deviations from normality were only detectable within the GC-biased *P. putida *genome. D'Agostino's K-squared test [28] identified an excess of genes having non-normal *SCB *null distributions (P < 0.05 for 340 of 5350 ORFs; 6.3%), although the skewness and kurtosis values were universally small (-1 × 10^-3 ^to 8 × 10^-4 ^and -6 × 10^-4 ^to 6 × 10^-4^, respectively) and the worst approximations were concentrated among genes with less than 100 degenerate codons (67 of 503 small ORFs being non-normal at P < 0.05).

### Prediction of gene expression levels

Using existing gene expression data, we examined the predictive power of several codon selection statistics and their robustness in the face of uncertainty regarding optimal parameterization. Here we considered those methods that rely on information about the frequency with which each codon is used within a set of ORFs optimized for translation (*f_o_*). A robust method will generate a consistently high level of performance when the *f_o _*table is constructed with any set of ORFs for which the codon usage has been biased by codon selection. We selected six datasets of transcript abundance data for evaluation: *E. coli *[29], *Pseudomonas aeruginosa *[30], *Bacteroides thetaiotaomicron *[31], *Bacillus anthracis *[32], *Saccharomyces cerevisiae *[33], and *Schizosaccharomyces pombe *[34]. These include both eukaryotes and bacteria from three phyla, with genomic nucleotide compositions ranging from strongly AT-biased to strongly GC-biased.

For each dataset, we examined the correlation of the transcript abundance data relative to each codon optimization statistic (CAI [21], GCB [23], ACE_u _[this study], Karlin's E [7], and MELP [17,18]) when the codon statistic was calibrated against the most abundant transcripts from the same dataset. Here, our intention is not to actually predict the transcript abundance data, but to evaluate the behavior of each method under optimal conditions. By calibrating with the dataset that the statistics are tested against, we avoid arbitrary decisions in parameterization that may inadvertently favor one method over another. To examine how each statistic responds to decreased precision in identifying the optimal genes, the number of genes contributing codons to *f_o _*was gradually increased, 20 at a time, until it included half of all genes (far more than would be used to construct *f_o _*for typical analyses). For the statistics that require an estimate of codon usage in the absence of codon selection (*f_n_*), we used the codon composition of the entire genome.

**Figure 1:**
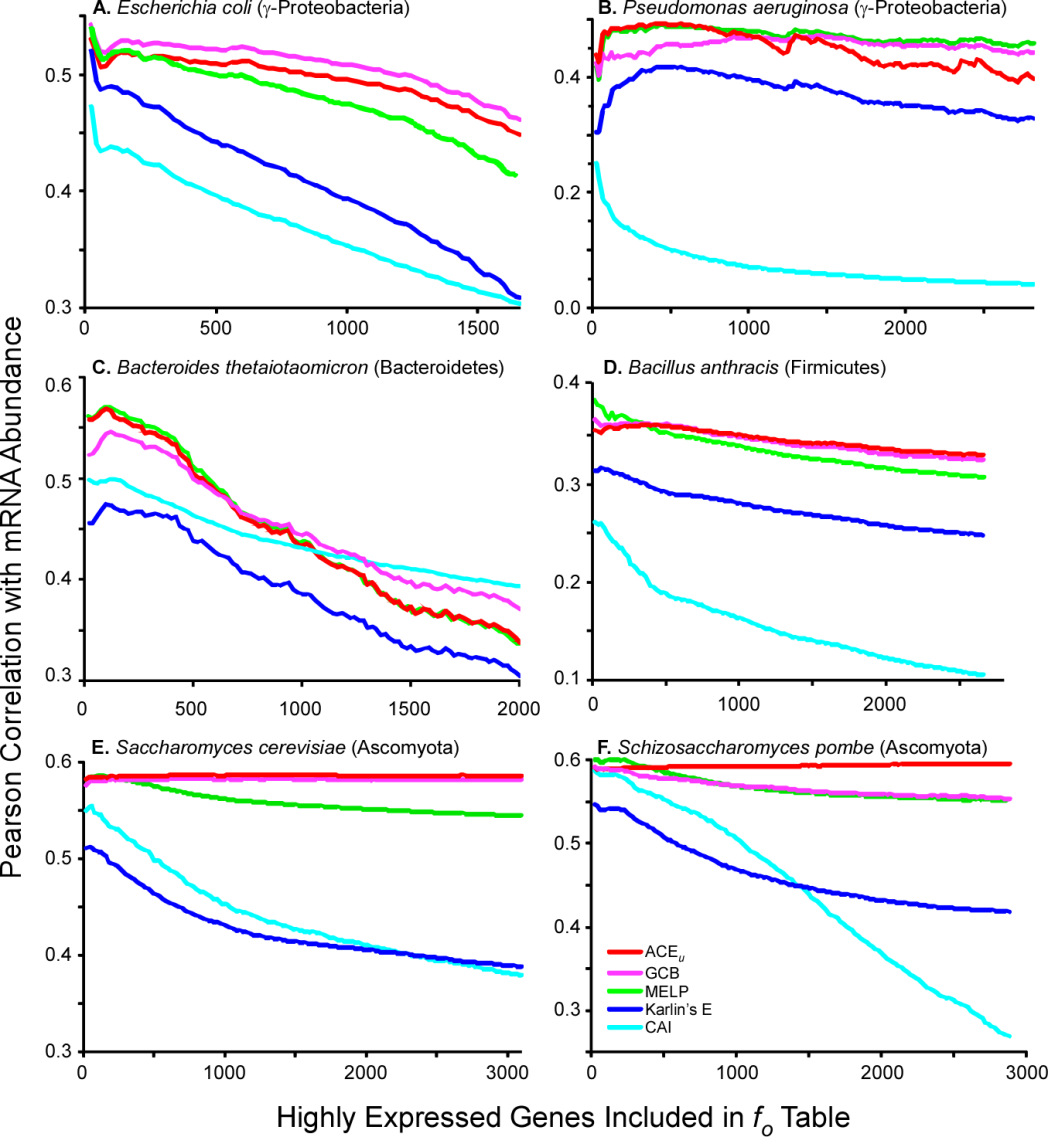
**Correlations coefficients of five different codon selection statistics with transcript abundance data (see text)**. The set of genes contributing to *f_o _*was systematically increased, 20 genes at a time, using the most highly expressed genes; typical *f_o _*tables use 5000-15000 codons. All ORFs were used to construct *f_n_*.

We observed substantial variation among codon bias statistics, with the highest correlations typically being produced by the ACE_u_,GCB, and MELP (Figure 1, Additional file 1 Figure S1), and these correlations being more robust to the decreased resolution of "highly expressed genes". Generally, CAI had the weakest correlation with expression level, particularly for *P. aeruginosa *(Figure 1B), which is expected given that this genome exhibits a strong bias in nucleotide composition (67% GC) and CAI does not incorporate any information about this bias [35].

The ability of the ACE_u _to predict gene expression levels in *P. aeruginosa *with such high accuracy (R = 0.65, 5543 genes, using the 100 most highly expressed genes to construct *f_o_*) is surprising in light of previous studies suggesting that there is little codon selection acting in this genome [8]. Grocock and Sharp [35] suggested that codon variation in *P. aeruginosa *was primarily due to the presence of genes with atypical nucleotide composition (presumably recently acquired), with a secondary trend due to codon selection. Recently acquired genes tend to be expressed weakly during growth in rich media, so that even in the absence of codon selection, a statistic that simply discriminated between native and foreign genes would be expected to correlate with expression levels. We tested whether this factor contributed to the high correlation by limiting the analyses to the 1678 genes that are likely to be native to *P. aeruginosa *because orthologs were detected in each of four other diverse *Pseudomonas *species: *P. mendocina, P. stutzeri, P. entomophila*, and *P. putida *(mean dS > 1.25 for each of the 10 pairs, where dS is synonymous divergence estimated by the method of [36]). For the 1677 genes in this set that also had transcript abundance values, the correlation coefficient actually increased to R = 0.75 using the same *f_o_*, indicating that most of this correlation is indeed due to codon selection.

### Prediction of substitution rates

The degree of codon bias also correlates (inversely) with the degree of divergence among orthologs. This is typically attributed to different levels of purifying selection or different mutation rates resulting from different frequencies of transcription [24,37-39]. We estimated the between-species synonymous divergence (dS) for genes in *P. aeruginosa*, *B. subtilis*, *E. coli*, *Staphylococcus haemolyticus*, and *Lactobacillus gasseri *relative to orthologs in other genomes, and calculated the correlation between dS and each of the codon selection statistics, using the Translation40 genes for *f_o_*. The ACE*_u _*generally produced a stronger correlation than the CAI (native or log transformed), was very similar to the GCB, and was sometimes exceeded by Karlin's E and Supek's MELP, which incorporate the same information about the expected codon usage but are not monotonic functions of codon optimization (Table 2).

**Table 2:** Correlation of codon statistics with rates of sequence divergence.

			Pearson correlation of codon statistic with dS					
**Reference Genome^1 ^**	**Target Genome**	**Mean dS^2^**	**ACE*_u_***	**CAI**	**E**	**MELP**	**GCB**	**RF^3^**

*B. subtilis *168	*B. subtilis *W23	0.24	-0.20	-0.18	-0.25	-0.22	-0.20	0.12
*P. aeruginosa *PA01	*P. aeruginosa *PA7	0.43	-0.23	-0.18	-0.18	-0.22	-0.23	0.13
*E. coli *K12	*E. fergusonii*	0.5	-0.29	-0.30	-0.26	-0.22	-0.29	0.15
*L. gasseri*	*L. johnsonii*	0.87	-0.56	-0.48	-0.65	-0.60	-0.56	0.41
*S. haemolyticus*	*S. lugdensis*	0.95	-0.48	-0.45	-0.47	-0.47	-0.48	0.19
*E. coli *K12	*S. enterica*	0.98	-0.51	-0.49	-0.61	-0.57	-0.51	0.26
*B. amyloliquifaciens*	*B. subtilis *W23	1.04	-0.51	-0.44	-0.54	-0.52	-0.50	0.32
*P. aeruginosa *PA01	*P. mendocina*	1.17	-0.59	-0.14	-0.58	-0.60	-0.59	0.40

1. Genome from which codon bias statistics were calculated. All codon statistics were calculated using the Translation40 gene set to construct *f_o _*and all ORFs to construct *f_n_*.

2. Average divergence (dS) and correlation were measured among putative orthologs with dS < 1.5 between reference and target genomes.

3. Correlation to log(probability) of belonging to the core genome as classified by Random Forest classifier [11]; RF values were calculated from a forest of 1000 trees as reported by Supek *et al*. [11].

### Algorithms for creating reference sets of non-optimized genes

ACE statistics rely on an expectation of the codon frequencies that would be observed in the absence of codon selection. Other methods for quantifying codon selection share this requirement, and these frequencies are often estimated from the codon composition of the entire genome under the premise that the majority of genes experience little codon selection. Yet genome-wide codon usage tables will be influenced both by genes experiencing strong codon selection and by genes recently introduced by lateral transfer whose codon usage patterns do not reflect the mutational history of their current genome. Eliminating both of these gene sets from this reference table should produce better predictions of gene expression data from codon frequency data. To exclude these classes of genes, we removed compositionally atypical genes, identified as those with dinucleotide or codon usage patterns that were maximally different from genome-wide averages [40,41]; as expected, this process excluded genes with extreme CAI values or atypical GC compositions at third codon positions (Additional file 2, Figure S2). Using systematically smaller subsets of *E. coli *genes to estimate *f_n_*, we saw improvement in the correlation between mRNA expression levels and ACE*_u_*, MELP, GCB and E (Additional file 3, Figure S3). The optimal reference table was reached when the most atypical ~30% of genes were excluded; additional reduction in the size of the set did not result in significant improvement.

For genomes lacking expression data for calibration, we developed an algorithm to identify a reasonable set of typical genes. Based on the assumption that removal of the most extreme 1% of genes produces more accurate δvalues, the algorithm continues to decrease the gene set by 1% increments as long as a significant majority of codons' δ values shift in the same direction as initially observed (P < 0.05; binomial test with expectation of 0.5). For *E. coli*, this resulted in a reference table constructed from 77% of the genes (Additional file 4, Figure S4), which is among the largest sets of compositionally typical, native genes that produced stronger correlations to expression data (Additional file 3, Figure S3). Therefore, this method provides a robust approach to selecting a less-biased and less noisy set of genes to approximate codon usage patterns produced by genome-wide processes alone.

### Algorithms for creating reference sets of selected genes

The ACE, like other methods, compares each gene's codon usage to the codon usage of a reference set of genes believed to have experienced strong codon selection (*f_o _*above). This set can be assembled by choosing genes that are known empirically to be highly expressed during rapid growth. However, these data are both biased to the laboratory conditions under which the organism is cultured and unavailable for many organisms. To eliminate these constraints, we used a two-step method to create this reference set from genomic data alone. To create an initial *f_o _*set, we selected a set of genes that could reasonably be inferred to have experienced codon selection; we examined such criteria as strong tAI [22], high χ^2 ^of codon usage [42], high values of the P2 metric [43], low values of ENC [15] or ENC' [16], atypical codon composition [7], homology to genes encoding the translation apparatus (*i.e*. Translation40), or strong conservation of amino-acid sequence in one or more genomes. Second, we iteratively selected an optimized gene set for each genome. Genes with the highest ACE*_u _*values were selected to create the *f_o _*table for the next round of the iteration. The processes began by selecting the most biased 40% of genes and reduced this set over 15 iterations until the final table size (10000 codons) was reached and the *f_o _*tables stabilized; this approach is similar to those used elsewhere, but based on different statistics [23,44].

Throughout this iteration process, codon scores are adjusted for the genome-wide tendencies, so the iteration algorithm identifies those genes that most exemplify the broad trend revealed when the initial parameterization set was compared to the whole genome set. Consequently, selection of the initial set is of utmost importance. Initial data sets, each generated using a different criterion, led to identical or nearly-identical reference sets after iteration for the 30 genomes that we examined (Additional file 5, Table S1). The most robust results came from initial reference sets comprised of genes encoding ribosomal proteins (as found elsewhere [17]), or genes which were most strongly conserved in the largest number of target genomes as determined by BLAST analysis (Additional file 5, Table S1); in both cases, biologically plausible reference sets - as determined by the genes' likely functions in the cell - were reached in >95% of genomes tested. In addition, the other methods, especially the tAI and P2 metrics, also converged on this same set of genes in most cases (Additional file 5, Table S1). The common iteration endpoint reached by multiple initial gene sets lends confidence that the final, iterated *f_o _*table is accurately reporting codon selection. As expected, the iterated *f_o _*table was similar to the Translation40 set of translation genes in most bacterial genomes.

Our ability to reach this endpoint without specifying particular genes sets (*e.g*., ribosomal proteins) allows the method to be extended to genomes of bacteriophages and other entities wherein highly-expressed genes are more difficult to identify *a priori*. For example, a similar analysis of the bacteriophage λ genome identified genes encoding structural proteins as those under strongest codon selection, and dispensable genes of the Nin region as those under the least selection (Additional file 6, Table S2). We examined the optimal size for the *f_o _*table by comparing the ACE*_u _*obtained with *f_o _*tables containing different numbers of codons against the mRNA transcript levels of those genes in *E. coli *and *P. aeruginosa *[29,30,45]. The optimal table size (i.e. the table generating the highest correlation between ACE*_u _*and transcript abundance) was found to be between 5,000 and 10,000 codons (Additional file 7, Figure S5).

### Summarizing genomic codon selection

The intensity of codon selection varies between genomes and several approaches have been implemented to measure these differences [8,10,13,22,46,47]. These studies have found that codon selection -- along with the number of tRNA and rRNA genes -- increases in bacteria with faster growth rates, suggesting that codon adaptation is one of several genomic structures that minimize generation time under optimal growth conditions [9,24].

Unlike other measures of gene-level codon usage bias, the ACE_z _lends itself naturally to estimates of genome-wide codon selection. A χ^2 ^distribution is defined as the sum of the squares of samples from a standard normal distribution. Therefore, we can calculate a normalized χ^2 ^statistic for each genome - measuring the overall degree by which genes deviate from the genome-wide expectation- by calculating the average of the squared Z-scores for each gene *g*, as(11)

In the absence of codon selection, values should approach 1.0, where the codon usage of each gene is a random sample [28]. The Monte Carlo simulations described above confirmed that when all ORFs share the same codon composition, the ACE_z _distribution for the genome has a mean of zero and a variance of one, resulting in a normalized χ^2 ^of 1.0.

To validate the behavior of the ACEχ^2 ^on real genomes, we examined two genomes (*P. aeruginosa *and *E. coli*) that are known to exhibit substantial codon selection, and one (*Buchnera aphidicola*) that is believed to experience negligible codon selection [48]. *P. aeruginosa *is of special interest because Grocock and Sharp [35] demonstrated that highly expressed genes exhibit distinctive codon usage in this genome, but Sharp *et al*.'s [8] attempt to estimate the strength of codon selection on 40 translational proteins revealed no selection (S = -0.019). This was attributed to the fact that S is based on the codons for only four amino acids, which did not include codons that were enriched in the highly expressed genes of *P. aeruginosa *[8]. Because ACE incorporates information from all synonymous codons, this limitation should be avoided.

**Figure 2:**
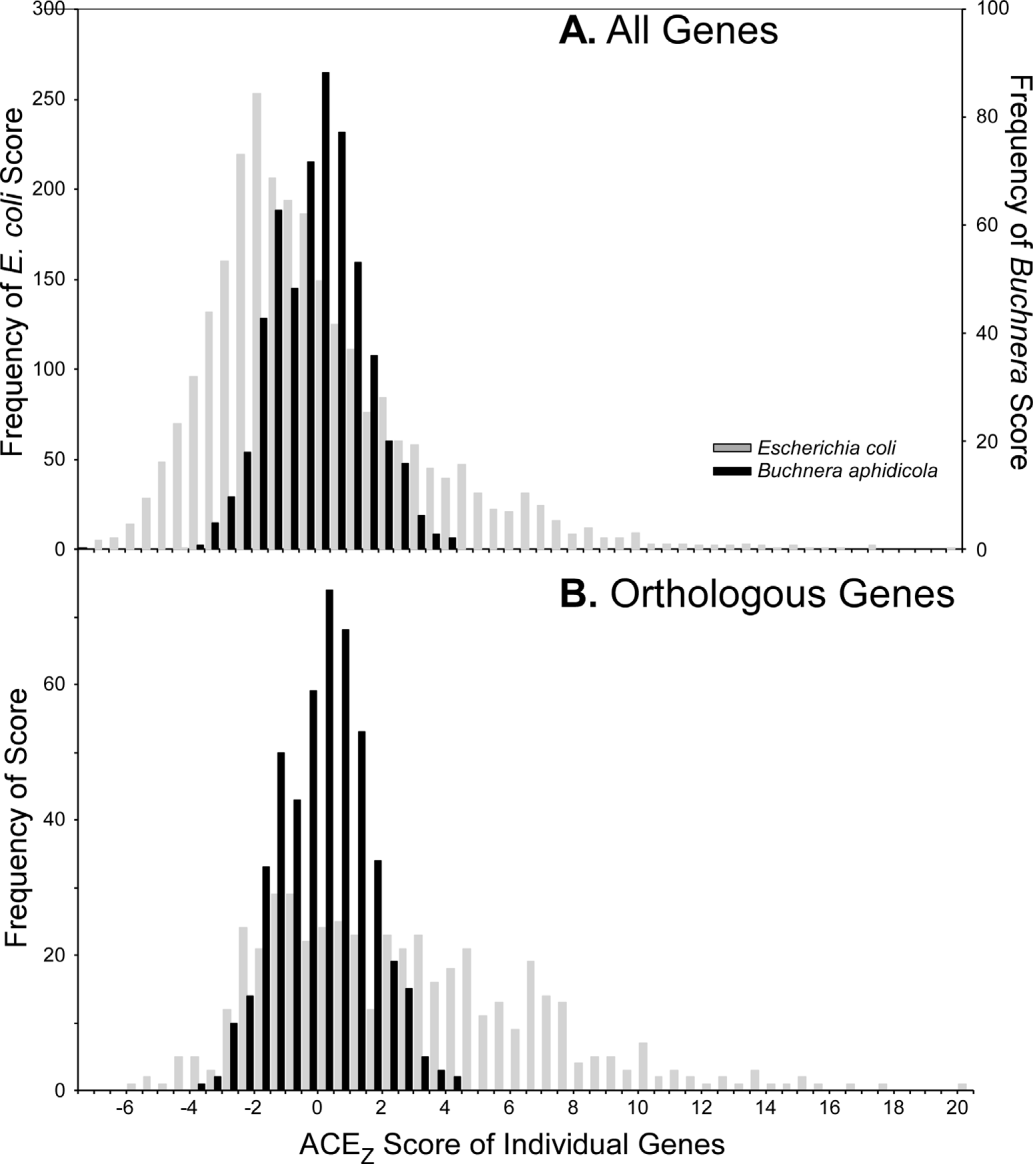
**Distribution of ACE*_z _*values for orthologs of *Escherichia coli *and *Buchnera aphidicola***. ACE*_z _*values were calculated using the Translation40 gene set to construct *f_o _*and all ORFs to construct *f_n_*. **A**. All genes from *E. coli *(4144 ORFs) and *B. aphidicola *(564 ORFs). **B**. Putative orthologs (499 ORFs) shared between *E. coli *and *B. aphidicola*.

The ACEχ^2 ^for the entire *P. aeruginosa *genome (5566 ORFs) was 3.7 when the Translation40 genes was used for *f_o_*, which is noticeably greater than the value expected in the absence of selection (Z ~ 88.9, P << 10^-10^). To test if ACEχ^2 ^is responding to codon usage variation that results from the inclusion of non-native genes, we limited the analysis (for both *f_n _*and ACEχ^2^) to the 1675 ORFs with orthologs in the four other *Pseudomonas *species; ACEχ^2 ^increased to 6.6 (Z ~ 75.4, P << 10^-10^). The great variation in ACEχ^2 ^values is illustrated by the *B. aphidicola *genome (564 ORFs), where ACEχ^2 ^was 1.97 when *f_o _*was calculated using the Translation40 genes (Table 3); in contrast, the ACEχ^2 ^for *E. coli *K12 (4144 ORFs) was 10.3. The differences in ACEχ^2^values between these genomes is not an artifact of comparing non-orthologous genes, since the *E. coli *genes that can be matched to *B. aphidicola *genes actually have higher ACE_z _values, and therefore would create an even greater ACEχ^2 ^value (Figure 2, Table 3).

**Table 3:** ACEχ^2 ^of three genomes calculated for different sets of genes.

	*Pseudomonas aeruginosa*	*Escherichia coli*	*Buchnera aphidicola*
All Genes	3.70 (5566)^a^	10.3 (4144)	1.97 (564)
Genus Core Genes	6.55 (1675)^b^	11.8 (2593)^d^	n/a
Enteric Core Genes	n/a^c^	28.7 (499)	1.94 (499)
Translation40 genes	72.6 (40)	63.6 (40)	4.63 (40)

a. ACE*_z _*values were calculated using the Translation40 gene set to construct *f_o _*and all ORFs to construct *f_n_*. Number of genes is reported in parentheses.

b. Putative orthologs of *Pseudomonas aeruginosa*, *P. mendocina, P. stutzeri, P. entomophila*, and *P. putida*.

c. Not applicable.

d. Putative orthologs of *E. coli*, *E. fergusonii*, and *E. albertii*

**Figure 3:**
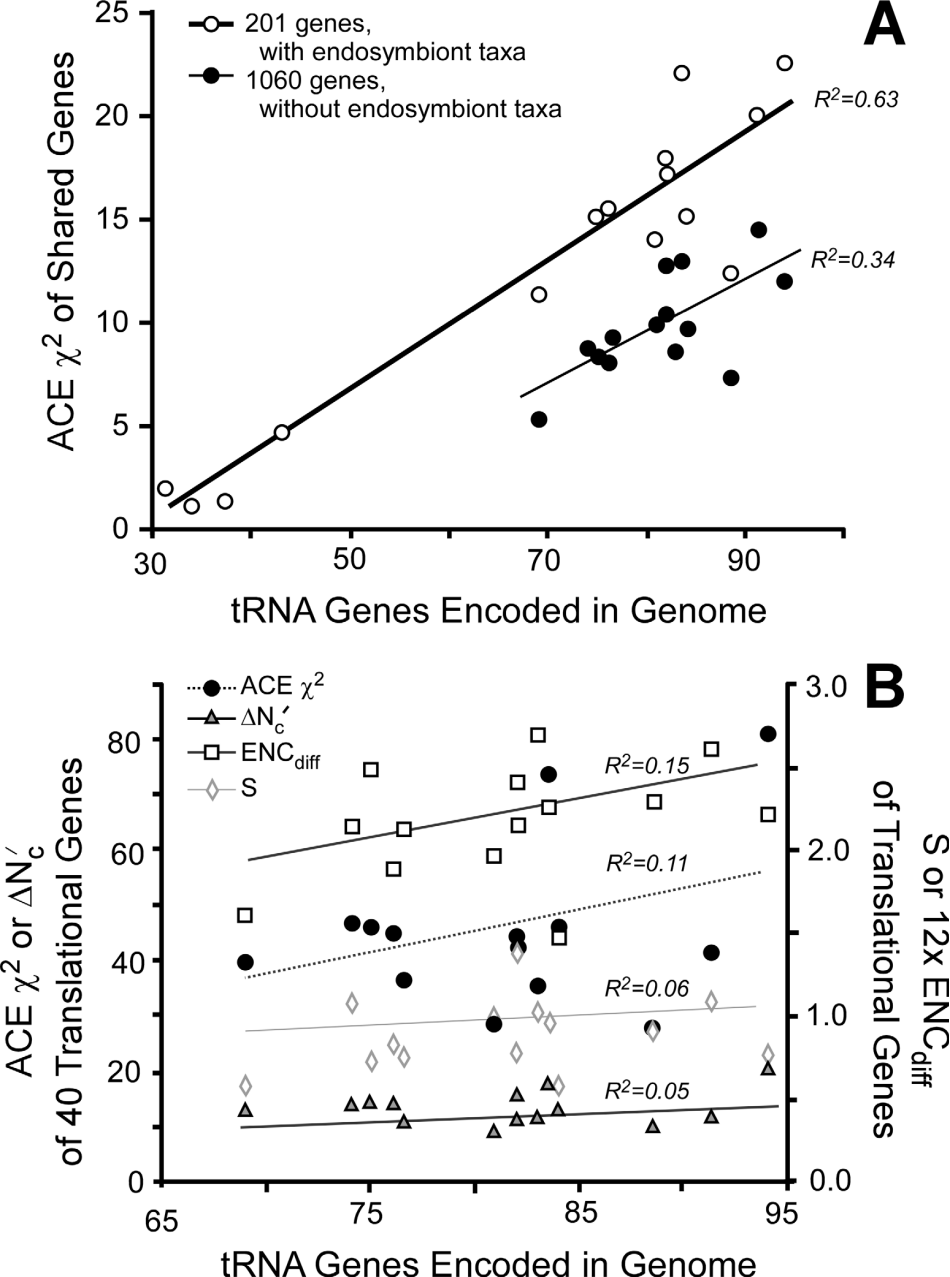
**Codon selection as a function of tRNA gene number in Enterobacteriaceae**. All codon statistics were calculated using the Translation40 gene set to construct *f_o _*and the shared set of ORFs to construct *f_n_***. A**. ACEχ^2 ^calculated on all shared genes, for the set of 14 free-living bacteria (1060 genes; open circles, thick line) and a set of 4 endosymbionts along with 11 free-living bacteria (201 genes; filled circles, thin line). **B**. ACEχ^2^, like other statistics, calculated on Translation40 gene set.

The contrast between endosymbiotic and free-living Enterobacteria was corroborated through comparison of four independent endosymbiont lineages [49,50] against eleven diverse free-living genomes. We selected 201 sets of putative orthologs present in each genome and, using the Translation40 genes to construct *f_o_*, calculated ACEχ^2 ^for these genes while using their combined codon composition as *f_n_*. These four endosymbionts had substantially lower ACEχ^2 ^than any of their free-living relatives (Figure 3A, Additional file 8 Table S3).

To test the sensitivity of the ACEχ^2 ^to genome-wide selection for efficient translation of highly expressed genes, we examined the correlation between ACEχ^2 ^and tRNA gene copy number, which is the complementary side of codon bias [8,10,46]. To assure a large sample of orthologous genes and minimize phylogenetic non-independence between genomes, we limited each analysis to a single bacterial family - the Enterobacteriaceae, Mycobacteriaceae, and Bacilliaceae (Table 4). Genomes (Additional file 9, Table S4) were selected such that no two genomes were separated by less than one substitution per synonymous site (dS), assuring that no two genomes were closely related and a sufficient number of mutational events had occurred such that meaningful differences in tRNA count and codon usage are possible. Unfortunately, we were unable to examine the relationship between codon optimization and maximal growth rate due to insufficient information about maximal growth rates in these groups [9].

**Table 4:** Correlation coefficients of genome-wide codon selection measures with the number of tRNA genes in each genome.

			Pearson Correlation Coefficient of tRNA Gene Number and Genome-wide Codon Selection Statistic^a^				
			
Family	Number of Genomes	Number of Orthologues	ACEχ^2^_core_^b^	ACEχ^2^_40_^b^	ENC_diff_	ΔN'_c_	S
Enterobacteriaceae	14	1060	0.628 (p = 0.008)^c^	0.338 (0.119)	0.264 (0.181)	0.399 (0.079)	0.257 (0.188)
Mycobacteriaceae	9	982	0.409 (0.167)	0.461 (0.106)	0.512 (0.080)	0.495 (0.088)	0.153 (0.347)
Bacilliaceae	12	541	0.556 (0.030)	0.440 (0.076)	0.373 (0.116)	0.346 (0.136)	0.427 (0.083)

a. All codon statistics were calculated using the Translation40 gene set to construct *f_o _*and the shared set of ORFs to construct *f_n_*.

b. ACEχ^2^_core _was calculated from all orthologues; ACEχ^2 ^40 was calculated from Translation40 genes.

c. Probability of calculating a correlation coefficient this large in the absence of a true correlation; one-tailed test using Fisher's z-transformed correlation.

To evaluate the Enterobacteriaceae without the influence of endosymbionts, we recalculated ACEχ^2 ^without the four endosymbionts mentioned above, but added three more genomes from free-living bacteria (Additional file 8, Table S3). Using the 1060 genes shared among these 14 genomes, we still detected a substantial correlation between tRNA gene number and ACEχ^2 ^(R^2 ^= 0.34; p = 0.01, one tailed test using Fisher's z-transformed correlation; Figure 3A). Noticeably, the relationship of ACEχ^2 ^among genomes is robust to the set of genes used to assign the *f_n _*frequencies; for the 11 genomes that were included in both analyses, the correlation of their values was 0.96, despite being calculated with 201 vs. 1060 genes. A significant correlation between ACEχ^2 ^and tRNA count is also seen for the Bacilliaceae (R^2 ^= 0.31; 12 genomes; p = 0.03), while the correlation in the Mycobacteriaceae (R^2 ^= 0.16; 9 genomes; p = 0.15) does not reach the standard significance cutoff of (p = 0.05) due to both a smaller correlation and smaller sample size (Table 4, Additional file 10 Table S5).

In contrast to the ACEχ^2^, other measures of codon selection detected little variation among genomes of non-endosymbiotic bacteria within the same family and none indicated a significant correlation with tRNA gene count (Table 4). We examined Dethlefsens and Schmidt's ΔN'_c _[46], Rocha's ENC_diff _[10], and Sharp's S [8], but excluded von Mandachs and Merkl's GCB_eff _[13]because it cannot be meaningfully calculated for many genomes. The Enterobacteriaceae contain the greatest number of sequenced genomes with a substantial amount of synonymous divergence among them (mean dS > 1.0 for all pairwise comparisons), so we focused on them for further investigation of the differences among the statistics (Figure 3B). A fundamental difference between the ACEχ^2 ^and other statistics is that ACEχ^2 ^examines the codon usage variation for a large portion of the genome (the core of shared genes in these examples), whereas the previously described methods examine how the codon usage frequencies of a specified subset of genes differs from the genome-wide average [8,10,46].

To test if this focus on the pre-selected set of optimized genes accounted for the differences between the statistics, we calculated ACEχ^2 ^for just the 40 genes that were used for the *f_o _*table, while continuing to use the 1060 shared genes for *f_n_*. This statistic (ACEχ^2^_40_) had only a moderate correlation to the ACEχ^2 ^for the shared genes (Additional file 10, Table S5), indicating that focusing on this smaller set of genes can substantially distort estimates of genome-wide codon selection. However, this is not the only explanation for the difference between the ACEχ^2 ^and other statistics, as the ACEχ^2^_40 _has an even weaker correlation to ENC_diff _and S, while being strongly correlated to ΔN'_c _(Additional file 10, Table S5).

## Discussion

### Interpretation of ACE

The ACE measures the effect of codon selection on codon usage, which is a slightly different concept than the magnitude of selection (*s*) described in population genetic theory. We have taken care to remove the influence of amino-acid composition from the ACE to provide a better prediction of physiological parameters such as gene expression levels. In contrast, an estimate of *s *should be sensitive to the amino acid composition, and a direct estimate of codon selection will likely provide better estimates of population diversity parameters such as the patterns of polymorphism [24]. Moreover, the ACE is a linear function of codon frequency; for an amino acid encoded by two codons, the contribution to ACE is directly proportional to the frequency of the preferred codon (P). In contrast, selection is a non-linear function of P (*i.e*. N_e_s = log[(kP)/(1-P)] where k represents the mutational balance), according to Sharp *et al*. [8].

The ACE uses an estimate of the codon composition specified as arising from genome-wide processes alone (*e.g*. mutation). We constructed a single table to reflect these codon frequencies, implicitly assuming that a uniform process is acting upon all genes in the genome. This assumption of mutational uniformity is less valid in some eukaryotic genomes that harbor isochores, but it is reasonable for bacteria once recently introduced genes are excluded. Slight violations of this assumption arise from subtle strand variation and origin-to-terminus gradients [37]. However, this variability does not generally affect calculation of ACE values; for example, while replication of Firmicutes involves distinct forms DNA polymerase III on each strand, leading to strong strand bias [51], the correlation between ACE values and dS does not improve when separate *f_n _*tables are created from leading and lagging strand genes (data not shown).

One final concern is that codons are not necessarily independent of their neighbors[52], or that synonymous sites may be constrained by functional demands aside from codon optimization for efficient translation. Among these constraints are determinants of chromosome architecture [53], mRNA structure [54], avoidance of ribosome-binding sites [55] or homopolymeric tracts [56], or even selection for the more slowly translated codon due to the kinetics of and protein synthesis [57].

### Variance in ACE

We modeled the stochastic distribution of the ACE as though each gene had a constant amino acid composition and each amino acid could be encoded by any of its cognate codons with a probability given by genome-wide substitution parameters. Of course, amino acids will vary stochastically in a constant regime of mutation and selection, and modeling such variation may increase the expected variance of the ACE, though the normalization across amino acids should minimize any variance introduced by amino acid substitutions. Regardless of that correction, amino acid composition can only crudely be modeled as a simple random variable because selective pressures acting on amino acid substitutions clearly are not uniform across the length of the protein.

Selection acting on synonymous substitutions varies among sites within ORFs [38,58-62]. The ACE is robust to this complication layered on top of the mutation-selection-drift model, and can be interpreted as being proportional to the number of sites under strong selection for use of the globally preferred codon. Such variation in the strength of selection among sites would reduce the stochastic variance in the ACE and other codon bias statistics.

### Identification of genome-wide influences on codon usage

The construction of two different codon frequency tables (*f_o _*and *f_n_*) allows us to separate the genome-wide influences on codon usage from codon selection, which has the greatest effect on highly expressed genes, causing *f_o _*to deviate from *f_n_*. The use of *f_n _*to normalize the iteration process avoids identifying a set of genes that represent the "dominating codon bias" [44], instead identifying a set representing codon selection [23]. The accuracy of *f_n _*has an important role in any analysis of codon selection. We demonstrated a method for identifying a set of genes that best represents the patterns of codon usage that would exist in the absence of codon selection. The codon usage in such genes is generally assumed to reflect mutational biases, but they may in fact be influenced by genome-wide selection for nucleotide composition or biased gene conversion [63-65]. Such complications would not affect the suitability of these genes to represent codon usage in the presence of minimal codon selection.

### Comparisons of codon selection among genes

The statistical framework of the ACE facilitates comparisons among genes within a genome. First, by accounting for the codon frequencies that are expected to be observed in the absence of codon selection, the ACE avoids spurious differences that can result from variation in amino acid composition among genes. A more fundamental difference between ACE and other statistics in this context is that the stochastic variation (*i.e*. sampling error) in ACE is approximately normally distributed, and its variance can be calculated despite each amino acid contributing a different amount of variation. For a given gene, the error variance of the ACE can be estimated as the variance that would occur in the *SCB *(Equation 7) when the expected codon frequencies are equal to the observed codon frequencies in that gene. The error variance of the ACE*_u _*is then the error variance of the ACE divided by the square of the denominator in Equation 10.

Having an estimate of the error variance, and knowing that the variance is approximately normally distributed, we are able to compare genes using the *t*-test for two independent samples [28]. This provides a statistical test for whether genes experience different degrees of codon selection. For example, the ACE*_u _*values for the independently transcribed methionine biosynthetic genes in *E. coli *range from -0.094 to -0.018 for the *metABC *genes to 0.10-0.14 for the *metEH *genes. While the ACE*_u _*of the *metABC *genes are not significantly different from each other (P > 0.2), all three are significantly different from those of the *metE *or *metH *genes (P < 0.01). This difference is not surprising as the *metABC *genes are only expressed during methionine starvation whereas the *metEH *genes also function to recycle S-adenosyl-homocysteine, a function that is required even during periods of methionine excess. Therefore, the significant difference in ACE*_u _*values supports the hypothesis that the *metEH *genes would be expressed under a larger number of growth conditions and, as a result, experience greater codon selection.

### Comparisons of codon selection across genomes

The ACEχ^2 ^is fundamentally different from previous attempts to quantify variation in the strength of codon selection between genomes. Three recently proposed methods have focused on a small fraction of the ORFs in each genome (*e.g*. ribosomal proteins) and used the deviation of their codon usage from the genome-wide average as an estimate of the efficacy of selection in each genome [8,10,46]. They interpret the strength of selection on a particular subset of ORFs as being representative of, or proportional to, the strength of selection acting on all ORFs in the genome. In contrast, ACEχ^2 ^can be calculated from all genes believed to be long-term residents of the genome. This greater inclusiveness may account for the fact that the ACEχ^2 ^generally correlates more strongly with the tRNA gene number than the other measures. The biological basis for the correlation between tRNA gene number and measures of codon selection remains unclear [66-68], and the ability of the ACEχ^2 ^to quantify codon selection across large sets of genes may facilitate investigations of this relationship.

### Extension of the ACE framework to other analyses

A strength of the ACE framework is its null model, which allows rigorous statistical tests to be applied to ACE*_z_*, ACE*_u _*and ACEχ^2^. This framework can be extended to other metrics. For example, the CAI has inspired other measures of codon usage bias, such as the tAI [22] and the eAI [69]. These statistics rely on scoring table values (*i.e.*, δ_ij_) that are derived from theories of how selection acts on the translational process, rather than being inferred from observed gene sequences. Despite this difference, these statistics are still amenable to the statistical tools developed for ACE, which may provide greater precision to the estimates of codon selection when investigating the molecular nature of codon selection (*e.g*. [70]).

## Conclusions

We have presented a statistical framework for the interpretation of codon usage biases in microbial genomes, both within and between genomes. The proposed summary statistic for quantifying variation within a genome incorporates the strengths that were previously only found in separate statistics, furthermore this work incorporates an analytical description of the sampling variance for the statistic. The methods presented here can also be applied to genomes for which we do not have prior information about gene expression and codon selection.

## Methods

### Sets of highly expressed genes for *f_o_*

Pre-selected sets of highly expressed were taken from previous literature. The set of 40 ribosomal proteins and translation elongation factors (Translation40, [8]) included the genes *tufA, tsf, fusA, rplA-rplF, rplI-rplT *and *rpsB-rpsT*. The codon count for each gene ignores the start codon. The values of *f_o _*are the count of each codon divided by the total count of codons for the same amino acid. If any codon is absent in the set of highly expressed gene, it is assigned a count of 0.5.

### Genomes used

All genome sequences were downloaded from the NCBI; genes were extracted from the primary (*i.e*. largest) replicon using the annotations provided by the RefSeq project. For genomes mentioned in the text, accession numbers appear in Additional file 9, Table S4.

Counts of tRNA genes can vary substantially among closely related genomes, so an average value was estimated for each species. Counts from each genome were made from the list of structural RNAs between 60 and 100 bp long, excluding the Sec tRNA. A species average was calculated using weights proportional to branch lengths on a tree constructed with MrBayes using 16S rRNA genes. The resulting values are close to the unweighted average of all genomes from that species in the NCBI database.

### Ortholog identification

Annotated open reading frames were translated and used as BLASTP queries to search databases composed of ORFs from each of the other genomes (e < 1) followed by semiglobal alignment. Sets of putative orthologs were assembled from those ORFs where each was a reciprocal best match with the others. For each analysis, a minimum amino acid similarity was enforced, with decreasing stringency for groups bearing more-divergent taxa: *Escherichia *85%; *Pseudomonas*, *Lactococcus*, *Mycobacterium*, *Staphylococcus *70%; Enterobacteriaceae, Bacilliaceae 60%.

### Codon statistics

Slight modifications were made to the described codon statistics to make them comparable with each other. The GCB, like the other statistics, was calculated without consideration of the stop codon. For the calculation of Pearson's correlation coefficient, a logarithmic transformation was applied to the transcript abundance, E [7], MELP [17,18], RF P-value [11] and CAI [21]. This generally increased the correlation between the transcript abundance and the codon bias statistics. Furthermore, it made the statistics conceptually comparable because the GCB [23] and ACE_u _are intrinsically calculated with logarithms. Spearman (rank) correlation coefficients were typically weaker and were not used.

### Software used

All analyses were performed with DNA Master, available at http://cobamide2.bio.pitt.edu, except where other software packages are explicitly mentioned in the text.

## Authors' contributions

ACR and JGL carried out the experiments, analyzed the data and wrote the paper. Both authors have read and approved the final manuscript.

## Supplementary Material

Additional file 1 **Figure S1**. Spearman correlation coefficients of five different codon selection statistics with transcript abundance data (see text). The set of genes contributing to *f_o _*was systematically increased, 20 genes at a time, using the most highly expressed genes. All ORFs were used to construct *f_n_*. Click here for file

Additional file 2 **Figure S2**. Histograms show the distributions of genes' %GC of third codon positions (B,D,F) and CAI values (A,C,E) of *E. coli *genes. Data series show successively smaller sets of genes whereby the most aberrant genes - as measured by Karlin's dinucleotide frequencies (A,B), Karlin's B metric of codon usage bias (C,D), or both (E,F) - were eliminated. Click here for file

Additional file 3 **Figure S3**. Pearson's correlation of different codon bias metrics and *E. coli *mRNA abundance [29] as a function of the percentage of genes remaining in the set of genes used to construct the *f_n _*table. The Translation40 set of genes were used to construct the *f_o _*table. Gene sets were reduced by eliminating the most aberrant genes - as measured by Karlin's dinucleotide frequencies, Karlin's B metric of codon usage bias, or both. Click here for file

Additional file 4 **Figure S4**. Determination of optimal genome size for constructing the *E. coli f_n _*table by progressive enrichment for nonselected codons. Reduced genomes had successively smaller sets of genes whereby the most aberrant genes - as measured by Karlin's dinucleotide frequencies, Karlin's B metric of codon usage bias, or both - were eliminated. Codons' δ values are compared between those when 100% and 99% of genes are analyzed. In smaller subsets of genes, the probability of observing a similar direction of change is calculated by a binomial test with expectation of 0.5. Significant improvement in the *f_n _*table is seen when the P value for maintaining a similar change in δ values is low (P < 0.01); once this probability rises, δ values are changing randomly. Click here for file

Additional file 5 **Table S1**. Performance of different methods to choose initial set of genes experiencing strong codon selection. Click here for file

Additional file 6 **Table S2**. ACE_z _values of bacteriophage lambda genes. Click here for file

Additional file 7 **Figure S5**. Correlation between genes' ACE values and mRNA expression level [29,30,45] as a function of the size of the number of codons in the *f_o _*table. Different *f_o _*tables were created by iteration as described in the text; tables were successively reduced in size selecting genes with the most extreme ACE*_u _*values to construct the next table. Click here for file

Additional file 8 **Table S3**. Properties of 18 genomes from Enterobacteriaceae. Click here for file

Additional file 9 **Table S4**. Strains used for ACE comparative genome analyses. Click here for file

Additional file 10 **Table S5**. Correlation of whole-genome measures of codon selection with tRNA count and with each other. Click here for file
