# Supplementary figures and images for: Quantification of codon selection for comparative bacterial genomics
Source: BMC Genomics. 2011 Jul 25;12:374. doi: 10.1186/1471-2164-12-374 (PMC3162537; doi:10.1186/1471-2164-12-374)

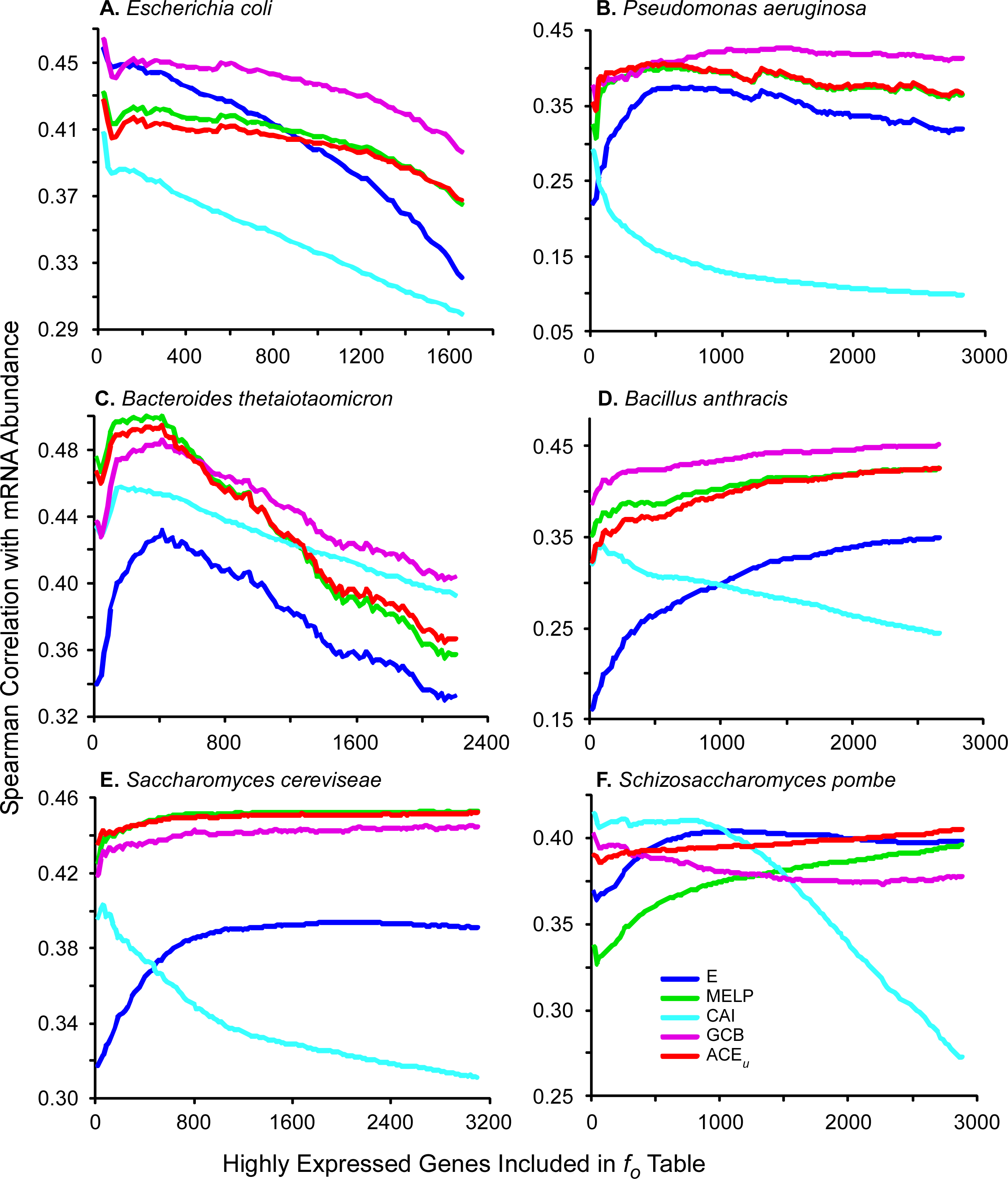

Supplement: Additional file 1 — Figure S1. Spearman correlation coefficients of five different codon selection statistics with transcript abundance data (see text). The set of genes contributing to fo was systematically increased, 20 genes at a time, using the most highly expressed genes. All ORFs were used to construct fn. [file 1471-2164-12-374-S1.TIFF]

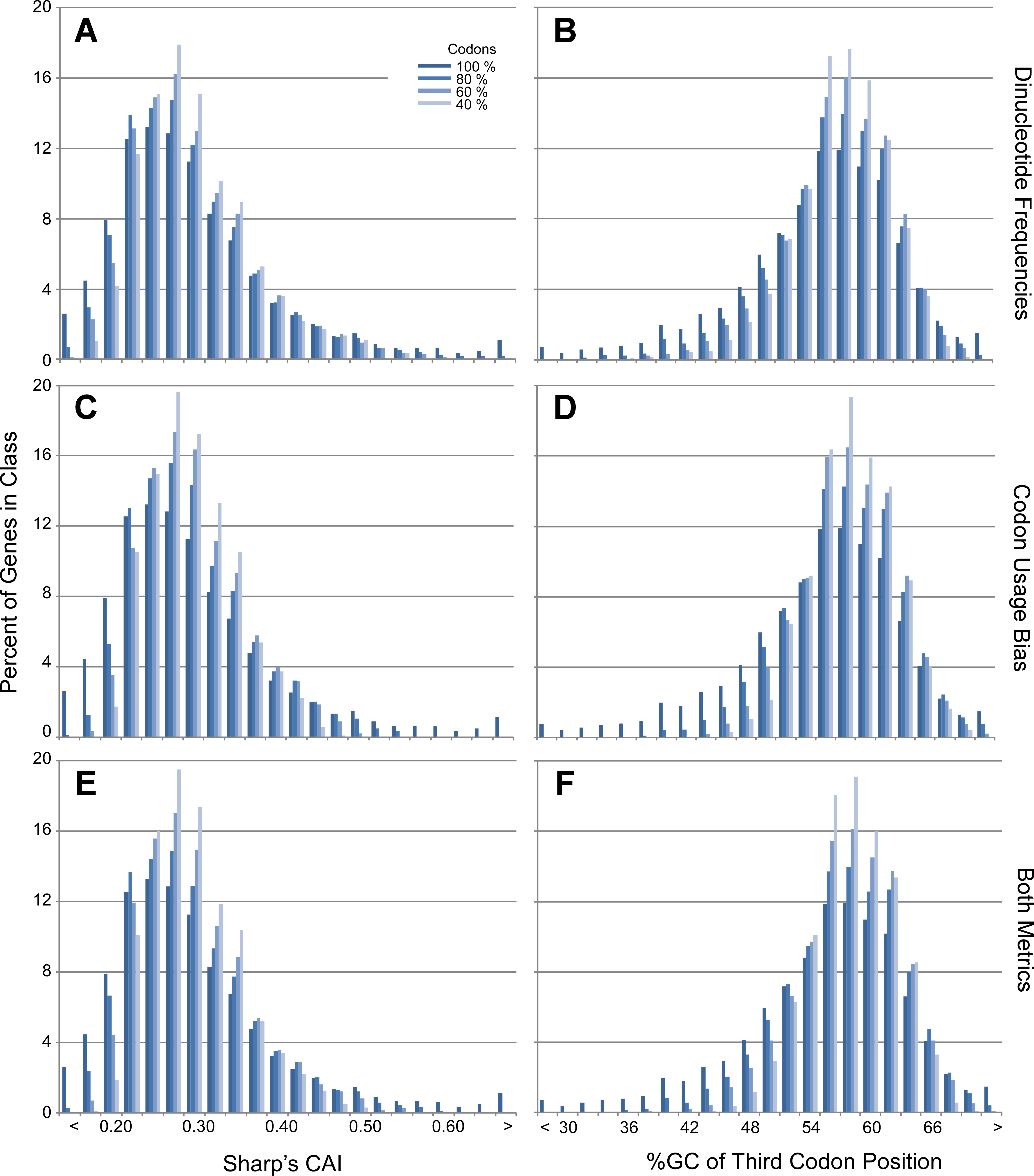

Supplement: Additional file 2 — Figure S2. Histograms show the distributions of genes' %GC of third codon positions (B,D,F) and CAI values (A,C,E) of E. coli genes. Data series show successively smaller sets of genes whereby the most aberrant genes - as measured by Karlin's dinucleotide frequencies (A,B), Karlin's B metric of codon usage bias (C,D), or both (E,F) - were eliminated. [file 1471-2164-12-374-S2.TIFF]

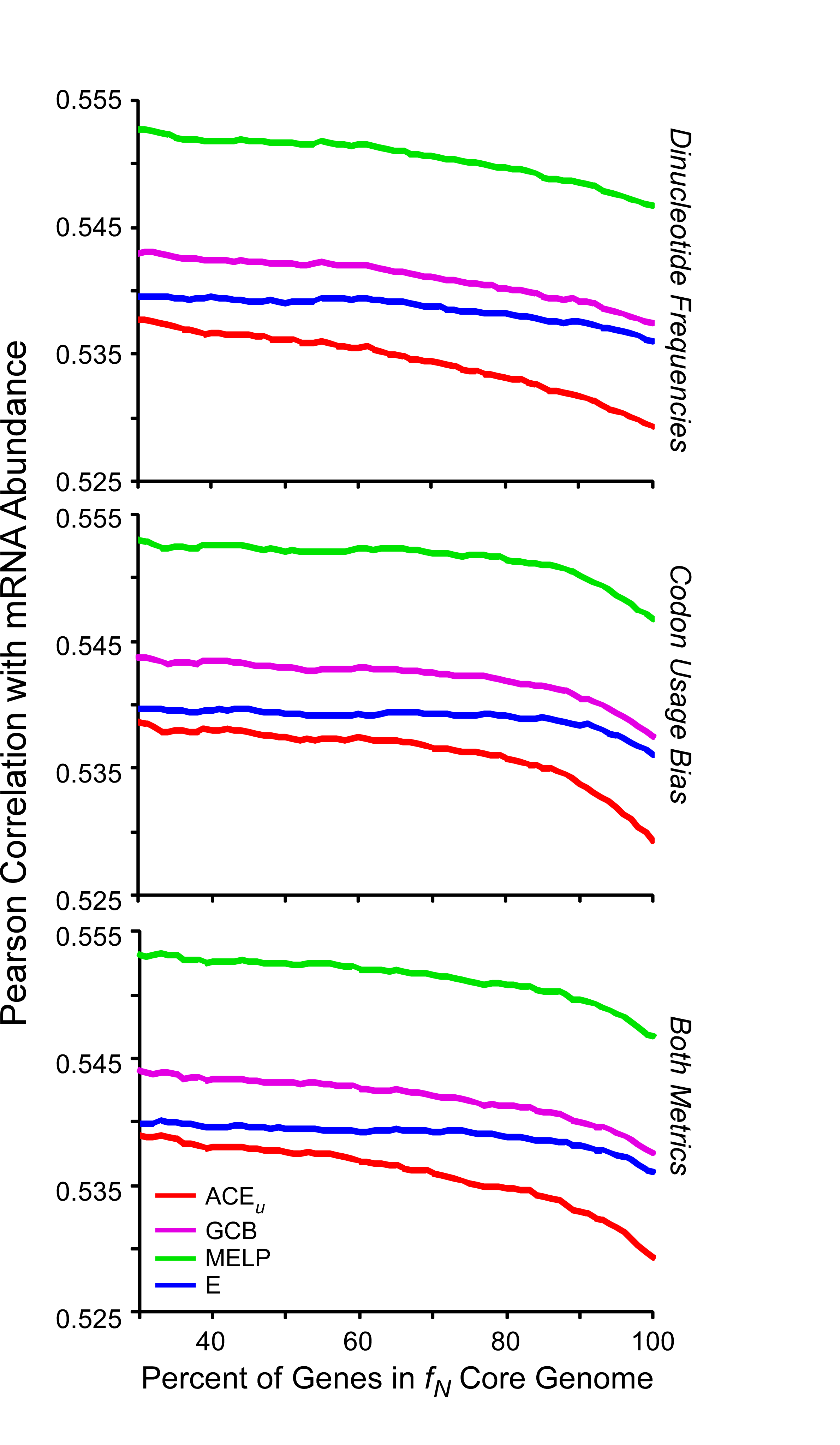

Supplement: Additional file 3 — Figure S3. Pearson's correlation of different codon bias metrics and E. coli mRNA abundance [29] as a function of the percentage of genes remaining in the set of genes used to construct the fn table. The Translation40 set of genes were used to construct the fo table. Gene sets were reduced by eliminating the most aberrant genes - as measured by Karlin's dinucleotide frequencies, Karlin's B metric of codon usage bias, or both. [file 1471-2164-12-374-S3.TIFF]

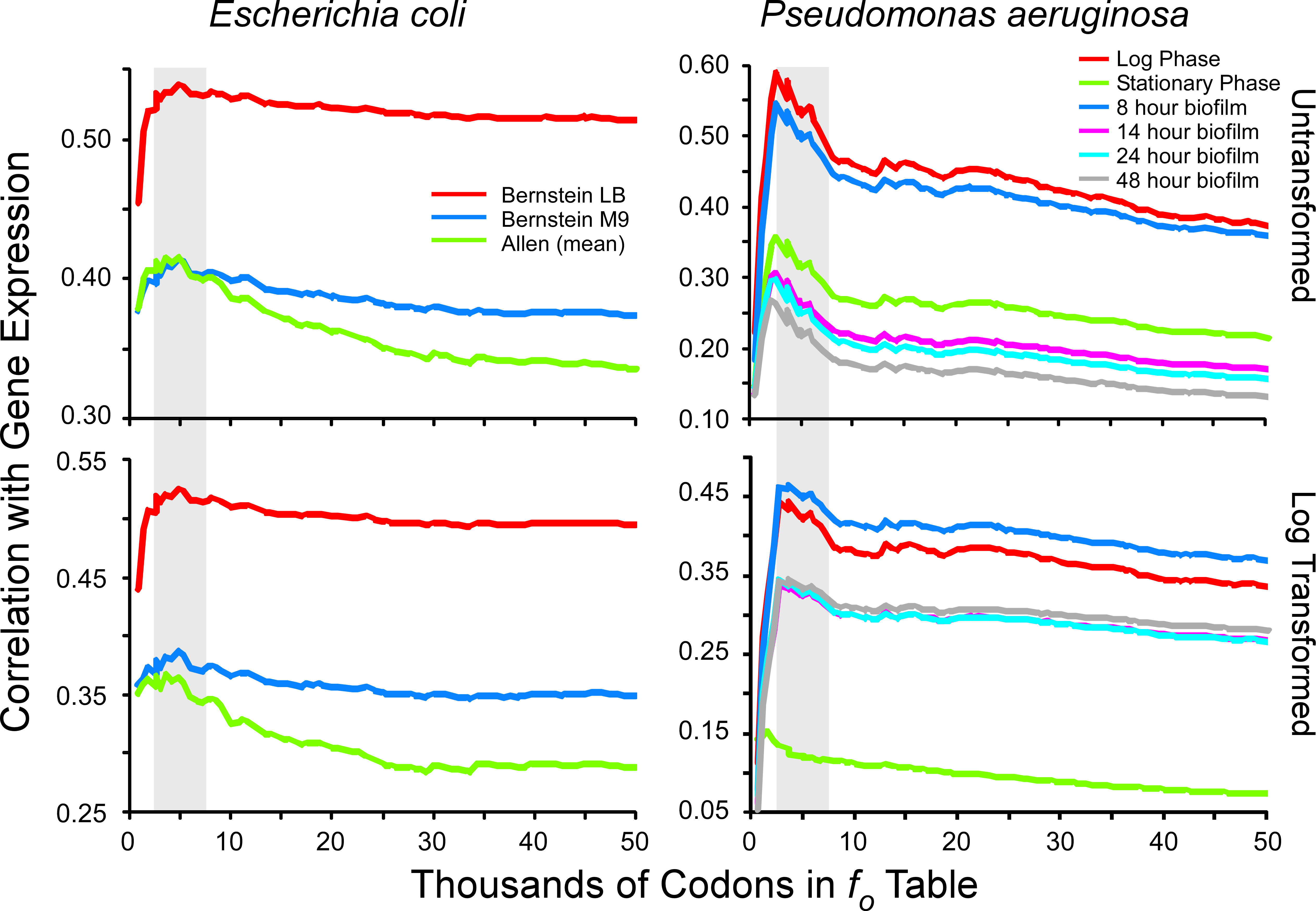

Supplement: Additional file 7 — Figure S5. Correlation between genes' ACE values and mRNA expression level [29,30,45] as a function of the size of the number of codons in the fo table. Different fo tables were created by iteration as described in the text; tables were successively reduced in size selecting genes with the most extreme ACEu values to construct the next table. [file 1471-2164-12-374-S7.TIFF]
