# Supplementary material for: Quantification of codon selection for comparative bacterial genomics
Source: BMC Genomics. 2011 Jul 25;12:374. doi: 10.1186/1471-2164-12-374 (PMC3162537; doi:10.1186/1471-2164-12-374)
Supplement: Additional file 5 — Table S1. Performance of different methods to choose initial set of genes experiencing strong codon selection. [file 1471-2164-12-374-S5.doc]

**Table S1.** Performance of different methods to choose initial set of genes experiencing strong codon selection.

| Genome | Accession | Division | %GC^a^ | Length^b^ | χ^2^ | P2 | ENC | ENC’ | B | tAI | BLAST | *rps/rpl* |
| --- | --- | --- | --- | --- | --- | --- | --- | --- | --- | --- | --- | --- |
| *Acidobacterium capsulatum* ATCC 51196 | NC_012483 | Acidobacteria | 60.50 | 4.13 | No^c^ | Yes | No | No | No | Yes | Yes | Yes |
| *Akkemansia muciniphila* ATCC BAA-835 | NC_010655 | Verrucomicrobia | 55.76 | 2.66 | No | Yes* | Yes | No | No | Yes* | Yes | Yes |
| *Anabaena variabilis* 29413 | NC_007413 | Cyanobacteria | 41.42 | 6.37 | Yes* | Yes* | No | Yes | No | Yes | Yes | Yes |
| *Aquifex aeolicus* VF5 | NC_000918 | Aquificae | 43.48 | 1.55 | Yes* | Yes | Yes | No | No | Yes | Yes | Yes |
| *Bacillus subtilis* 168 | NC_000964 | Firmicutes | 43.52 | 4.21 | Yes* | Yes | Yes | Yes | Yes | Yes | Yes | Yes |
| *Bacteroides fragilis* 9343 | NC_003228 | Bacteroidetes | 43.19 | 5.21 | Yes* | Yes | Yes* | Yes | No | Yes | Yes | Yes |
| *Borrelia burgdorferi* B31 | NC_001318 | Spirochettes | 28.17 | 0.91 | Yes* | No | Yes | No | No | Yes | Yes | Yes |
| *Campylobacter curvus* 525.92 | NC_009715 | ε-Proteobacteria | 44.54 | 1.97 | Yes* | Yes | No | Yes | Yes | No | Yes* | Yes |
| *Chlamydophila pneumoniae* TW-183 | NC_005043 | Chlamydiae | 40.58 | 1.23 | Yes* | Yes | No | Yes | Yes | Yes | Yes | No |
| *Chlorobium tepidum* TLS | NC_002932 | Chlorobi | 56.53 | 2.15 | No | Yes* | Yes* | No | No | Yes* | Yes | Yes |
| *Chloroflexus sp.* Y-400 fl | NC_012032 | Chloroflexi | 56.68 | 5.26 | Yes* | No | Yes* | No | No | No | No | Yes* |
| *Deinococcus radiodurans R1* | NC_001263 | Deinococcus-Thermus | 66.61 | 3.28 | No | Yes | Yes | No | No | Yes | Yes | Yes |
| *Desulfovibrio desulfuricans* G20 | NC_007519 | δ-Proteobacteria | 57.84 | 3.73 | No | No | Yes | No | No | Yes | Yes | Yes |
| *Dictyoglomus thermophilum* H-6-12 | NC_011297 | Dictyoglomi | 33.74 | 1.96 | Yes* | No | Yes* | No | No | No | Yes | Yes |
| *Escherichia coli* MG1655 | NC_000913 | γ-Proteobacteria | 50.79 | 4.74 | Yes* | Yes | Yes | Yes | Yes | Yes | Yes | Yes |
| *Frankia sp. CcI3* | NC_007777 | Actinobacteria | 70.08 | 5.43 | Yes | Yes | No | No | No | No | Yes | Yes |
| *Fusobacterium nucleatum* ATCC 25586 | NC_003454 | Fusobacteria | 27.15 | 2.17 | Yes* | Yes | Yes | No | No | Yes | Yes | Yes |
| *Gemmatimonas aurantiaca* T-27 | NC_012489 | Gemmatimonadetes | 64.27 | 4.64 | Yes* | Yes | Yes | No | No | Yes | Yes | Yes |
| *Haemophilus influenze* Rd KW20 | NC_000907 | γ-Proteobacteria | 38.15 | 1.74 | Yes* | Yes | Yes | Yes | Yes | Yes | Yes | Yes |
| *Mycobacterium tuberculosis* F11 | NC_009565 | Actinobacteria | 65.62 | 4.42 | No | No | Yes | No | No | Yes | Yes | Yes |
| *Mycoplasma agalactiae* PG2 | NC_009497 | Tenericutes | 29.71 | 0.88 | Yes | Yes | Yes | Yes | Yes | Yes | Yes | Yes |
| *Neisseria gonorrhoeae* FA 1090 | NC_002946 | β-Proteobacteria | 52.69 | 2.15 | No | Yes | Yes | No | No | Yes | Yes | Yes |
| *Prochlorococcus marinus* MIT9313 | NC_005091 | Cyanobacteria | 50.74 | 2.41 | No | No | Yes | No | No | Yes | Yes | Yes |
| *Pseudomoans aeruginosa* PAO1 | NC_002516 | γ-Proteobacteria | 66.56 | 6.26 | No | Yes | Yes* | No | No | Yes | Yes | Yes |
| *Rhodopirellula baltica* SH 1 | NC_005027 | Planctomycetes | 55.40 | 7.15 | No | Yes | Yes | No | No | Yes | Yes | Yes |
| *Rickettsia prowazeckii* Madrid E | NC_000963 | α-Proteobacteria | 29.00 | 1.11 | Yes* | Yes* | Yes* | No | No | No | No | Yes* |
| *Sinorhizobium meliloti* 1021 | NC_003047 | α-Proteobacteria | 62.73 | 3.65 | Yes* | Yes | Yes | No | No | Yes | Yes | Yes |
| *Thermodesulfovibrio yellowstonii* DSM11347 | NC_011296 | Nitrospirae | 34.13 | 2.00 | Yes* | Yes* | No | Yes* | No | No | Yes | Yes |
| *Thermotoga maritime* MSB8 | NC_000853 | Thermotogae | 46.25 | 1.86 | Yes* | Yes | Yes | No | No | Yes | Yes* | Yes |
| *Thermus thermophilus* HB27 | NC_005835 | Deinococcus-Thermus | 69.41 | 1.89 | No | Yes* | No | No | No | No | Yes* | Yes* |
| Overall Performance |  | 30 Genomes |  |  | 63% | 80% | 77% | 30% | 20% | 77% | 94% | 97% |

1. %GC of the major replicon
2. Length of the major replicon in MB
3. Metric used to select genes initially believed to experience strong codon selection. ‘Yes’ indicates that iteration produced an optimal codon usage table; ‘No’ indicates that the iteration failed to find an optimal table; ‘Yes*’ indicates that a variant of the optimal table, with only one or 2 genes being different, was found.
