# Supplementary material for: Quantification of codon selection for comparative bacterial genomics
Source: BMC Genomics. 2011 Jul 25;12:374. doi: 10.1186/1471-2164-12-374 (PMC3162537; doi:10.1186/1471-2164-12-374)
Supplement: Additional file 6 — Table S2. ACEz values of bacteriophage lambda genes. [file 1471-2164-12-374-S6.DOC]

**Table S2.** ACE_z_ values of bacteriophage lambda genes.

| Gene | Start | Codons | ACEz^1^ | Function |
| --- | --- | --- | --- | --- |
| J | 15505 | 1092 | 8.1584 | Tail tip |
| H | 10542 | 816 | 6.7593 | Tape Measure |
| B | 2836 | 501 | 5.4484 | Head |
| E | 6135 | 325 | 4.9349 | Major Capsid |
| A | 711 | 608 | 4.6332 | Terminase |
| L | 13429 | 223 | 3.7507 | Tail |
| K | 14276 | 187 | 3.7103 | Tail |
| G | 9711 | 132 | 3.6723 | Tail Chaperone |
| C | 4418 | 420 | 3.6383 | Head |
| Z | 7977 | 186 | 3.3841 | Tail |
| T | 10115 | 132 | 3.3262 | Tail Chaperone |
| Fi | 7202 | 129 | 3.2695 | DNA packaging |
| V | 8955 | 234 | 3.0443 | Major Tail |
| Fii | 7612 | 113 | 3.0025 | Head/Tail joining |
| nu1 | 191 | 176 | 3.0012 | Terminase |
| *lom* | 18965 | 195 | 2.8653 | Outer membrane |
| I | 14773 | 214 | 2.8245 | Tail |
| M | 13100 | 101 | 2.8121 | Tail |
| D | 5747 | 105 | 2.8002 | Head decoration |
| *exo* | 31348 | 210 | 2.7948 | Exonuclease |
| U | 8552 | 123 | 2.7511 | Tail |
| orf206b | 20147 | 205 | 1.9083 |  |
| Rz | 45966 | 148 | 1.5506 | Lysis |
| *bet* | 32025 | 249 | 1.2379 | Recombination |
| orf-194 | 21973 | 184 | 0.8636 | Tail fiber assembly |
| nu3 | 5132 | 194 | 0.8458 | Capsid assembly |
| orf28 | 34271 | 26 | 0.6717 |  |
| orf-401 | 19650 | 395 | 0.5702 | Tail fiber |
| P | 39582 | 219 | 0.3939 | Replication |
| Rz1 | 46186 | 52 | 0.2185 | Lysis |
| W | 2633 | 65 | 0.0743 | Head/Tail joining |
| *ren* | 40280 | 91 | 0.0715 | Dispensible |
| lambdap79 | 47738 | 64 | -0.1133 |  |
| orf61 | 30839 | 58 | -0.2552 |  |
| *gam* | 32816 | 132 | -0.367 | Recombination |
| NinF | 42269 | 53 | -0.5686 | Dispensible |
| ral | 34087 | 59 | -0.719 | Restriction alleviation |
| ea22 | 29847 | 176 | -0.7805 |  |
| R | 45493 | 151 | -0.8569 | Endolysin |
| orf-314 | 21029 | 307 | -0.9156 | Tail fiber |
| orf60a | 31169 | 58 | -1.0746 |  |
| *kil* | 33187 | 43 | -1.1204 |  |
| *cro* | 38041 | 64 | -1.2928 | Transcription Repressor |
| NinI | 43224 | 211 | -2.1944 | Dispensible |
| cII | 38360 | 89 | -2.2344 | Transcription Activator |
| *bor* | 46459 | 95 | -2.2567 |  |
| rexb | 35825 | 139 | -2.3303 | Exclusion |
| O | 38686 | 281 | -2.3512 | Replication |
| S | 45186 | 102 | -2.423 | Antiholin |
| S' | 45192 | 101 | -2.4434 | Holin |
| cIII | 33299 | 51 | -2.654 | Antitermination |
| NinG | 42429 | 196 | -2.6564 | Dispensible |
| NinD | 41950 | 52 | -2.7841 | Dispensible |
| NinE | 42090 | 57 | -2.8528 | Dispensible |
| N | 35037 | 129 | -2.8664 | Antiterminator |
| rexa | 36275 | 272 | -2.8942 | Exclusion |
| lambdap35 | 29118 | 50 | -3.0032 |  |
| Q | 43886 | 195 | -3.114 | Antiterminator |
| NinH | 43040 | 63 | -3.2194 | Dispensible |
| ea10 | 33536 | 113 | -3.4241 | ssDNA Binding |
| ea8.5 | 29374 | 88 | -3.5797 |  |
| *xis* | 28860 | 69 | -3.6257 | Directionality Factor |
| NinC | 41081 | 274 | -3.6434 | Dispensible |
| orf63 | 31005 | 59 | -3.8966 |  |
| cI | 37227 | 225 | -4.4414 | Transcription Repressor |
| ea31 | 24509 | 285 | -4.5673 |  |
| NinB | 40644 | 137 | -5.0144 | Dispensible |
| orf-64 | 44621 | 62 | -5.0229 |  |
| lambdap48 | 34482 | 172 | -5.0556 | Superinfection exclusion |
| *int* | 27812 | 344 | -5.0724 | Integrase |
| lambdap78 | 47042 | 172 | -6.3975 |  |
| ea59 | 25396 | 510 | -9.0013 |  |
| ea47 | 22686 | 391 | -10.3823 |  |

1. ACE*_Z_* values calculated using an *f_n_* table constructed from all genes and a 5000-codon *f_o_* table constructed using genes with maximal χ^2^ of codon usage as an iteration seed.
