# Supplementary material for: Quantification of codon selection for comparative bacterial genomics
Source: BMC Genomics. 2011 Jul 25;12:374. doi: 10.1186/1471-2164-12-374 (PMC3162537; doi:10.1186/1471-2164-12-374)
Supplement: Additional file 8 — Table S3. Properties of 18 genomes from Enterobacteriaceae. [file 1471-2164-12-374-S8.DOC]

**Table S3: Properties of 18 genomes from Enterobacteriaceae.**

| **Organism** | **tRNA^1^**  **Count** | **tRNA average^2^**  **(# genomes)** | | 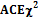  **(201 genes)** | 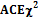  **(1060 genes)** | 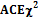  **(40 genes)** | **ENC_diff_** | **ΔN′_c_** | **S** |
| --- | --- | --- | --- | --- | --- | --- | --- | --- | --- |
| *Edwardsiella tarda* EIB202 | 94 | 94 | (2) | 22.7 | 12.0 | 88.3 | 0.192 | 22.4 | 0.817 |
| *Escherichia coli str*. K-12 substr. MG1655 | 85 | 91.3 | (29) | 20.1 | 14.5 | 47.5 | 0.232 | 13.9 | 1.19 |
| *Serratia proteamaculans* 568 | 84 | 84 | (1) | 15.3 | 9.8 | 51.7 | 0.132 | 14.8 | 0.652 |
| *Photorhabdus luminescens subsp. laumondii* TTO1 | 84 | 88.5 | (2) | 12.6 | 7.3 | 30.6 | 0.204 | 11.1 | 0.948 |
| *Enterobacter* *sp*. 638 | 82 | 82 | (3) | 17.3 | 12.8 | 48.0 | 0.190 | 13.0 | 0.846 |
| *Proteus* *mirabilis* HI4320 | 82 | 82 | (1) | 18.1 | 10.3 | 48.6 | 0.211 | 17.6 | 1.47 |
| *Xenorhabdus* *bovienii* SS-2004 | 83 | 83 | (1) | *N/A* | 8.6 | 39.6 | 0.237 | 13.2 | 1.09 |
| *Cronobacter* *sakazakii* ATCC BAA-894 | 82 | 83.5 | (2) | 22.2 | 13.0 | 82.5 | 0.196 | 19.6 | 1.02 |
| *Erwinia* *tasmaniensis* Et1/99 | 82 | 76.1 | (6) | 15.7 | 8.2 | 49.0 | 0.166 | 15.7 | 0.88 |
| *Yersinia enterocolitica subsp. enterocolitica* 8081 | 80 | 80.9 | (6) | 14.2 | 9.9 | 32.9 | 0.172 | 10.8 | 1.06 |
| *Pectobacterium* *atrosepticum* SCRI1043 | 76 | 76.6 | (3) | *N/A* | 9.3 | 40.0 | 0.186 | 12.3 | 0.810 |
| *Dickeya* *dadantii* Ech703 | 74 | 75 | (2) | 15.3 | 8.4 | 49.8 | 0.215 | 15.7 | 0.741 |
| *Sodalis* *glossinidius* str*.* 'morsitans' | 69 | 69 | (1) | 11.5 | 5.3 | 41.9 | 0.140 | 14.0 | 0.625 |
| *Pantoea ananatis* LMG 20103 | 67 | 74.1 | (3) | *N/A* | 8.8 | 50.82 | 0.186 | 15.1 | 1.13 |
| *Hamiltonella defensa* 5AT (*Acyrthosiphon pisum*) | 43 | 43 | (1) | 4.9 | *N/A* | *N/A* | *N/A* | *N/A* | *N/A* |
| *Blochmannia pennsylvanicus* str. BPEN | 39 | 37.3 | (3) | 1.6 | *N/A* | *N/A* | *N/A* | *N/A* | *N/A* |
| *Wigglesworthia glossinidia* (*Glossina* *brevipalpis*) | 34 | 34 | (1) | 1.4 | *N/A* | *N/A* | *N/A* | *N/A* | *N/A* |
| *Buchnera aphidicola* str. APS | 31 | 31.2 | (6) | 2.2 | *N/A* | *N/A* | *N/A* | *N/A* | *N/A* |

1. The Number of tRNAs in the genome analyzed for codon usage bias (tRNA^Sel^ genes are ignored when present)
2. The average number of tRNAs in the species; the number of genomes analyzed is given in parentheses. The tRNA count values for each genome were weighted according to the Bayesian phylogeny of the respective rRNA gene
