# Supplementary material for: Quantification of codon selection for comparative bacterial genomics
Source: BMC Genomics. 2011 Jul 25;12:374. doi: 10.1186/1471-2164-12-374 (PMC3162537; doi:10.1186/1471-2164-12-374)
Supplement: Additional file 9 — Table S4. Strains used for ACE comparative genome analyses. [file 1471-2164-12-374-S9.DOC]

**Table S4**. Strains used for ACE comparative genome analyses.

| Genome | Accession |
| --- | --- |
| ***Pseudomonas* species** |  |
| *P. aeruginosa* PAO1 | NC_009565 |
| *P. aeruginosa* PA7 | NC_009656 |
| *P. entomophila* L48 | NC_008027 |
| *P. mendocina* ymp | NC_009439 |
| *P. putida* KT2440 | NC_002947 |
| *P. stutzeri* A1501 | NC_009434 |
| **Mycobacteriaceae** |  |
| *Mycobacterium abscessus* ATCC 19977 | NC_010397 |
| *M. avium* 104 | NC_008595 |
| *M. gilvum* PYR-GCK | NC_009338 |
| *M. leprae* TN | NC_002677 |
| *M. smegmatis* str. MC2 155 | NC_008596 |
| *Mycobacterium sp*. JLS | NC_009077 |
| *M. tuberculosis* H37Rv | NC_000962 |
| *M. ulcerans* Agy99 | NC_008611 |
| *M. vanbaalenii* PYR-1 | NC_008726 |
| **Bacilliales** |  |
| *Anoxybacillus flavithermus* WK1 | NC_011567 |
| *Bacillus cereus subsp. cytotoxis* NVH 391-98 | NC_009674 |
| *B. clausii* KSM-K16 | NC_006582 |
| *B. halodurans* C-125 | NC_002570 |
| *B. megaterium* DSM319 | NC_014103 |
| *B. pseudofirmus* OF4 | NC_013791 |
| *B. selenitireducens* MLS10 | NC_014219 |
| *B. subtilis subsp. sub*tilis str. 168 | NC_000964 |
| *B. subtilis subsp. spizizenii* str. W23 | NC_014479 |
| *Geobacillus* sp. WCH70 | NC_012793 |
| *G. thermodenitrificans* NG80-2 | NC_009328 |
| *Lactobacillus gasseri* ATCC 33323 | NC_008530 |
| *L. johnsonii* FI9785 | NC_008530 |
| *Lysinibacillus sphaericus* C3-41 | NC_010382 |
| *Oceanobacillus iheyensis* HTE831 | NC_004193 |
| *Staphylococcus haemolyticus* JCSC1435 | NC_007168 |
| *S. lugdunensis* HKU09-01 | NC_013893 |
| **Enterobacteriaceae** |  |
| *Blochmannia pennsylvanicus* str. BPEN | NC_007292 |
| *Buchnera aphidicola str. APS* (*Acyrthosiphon pisum*) | NC_002528 |
| *Cronobacter sakazakii* ATCC BAA-894 | NC_009778 |
| *Dickeya dadantii* Ech703 | NC_012880 |
| *Edwardsiella tarda* EIB202 | NC_013508 |
| *Enterobacter* sp. 638 | NC_009436 |
| *Erwinia tasmaniensis* Et1/99 | NC_010694 |
| *Escherichia albertii* TW07627 | NZ_ABKX00000000 |
| *E. coli* str. K-12 MG1655 | NC_000913 |
| *E. fergusonii* ATCC 35469 | NC_011740 |
| *Hamiltonella defensa* 5AT (*Acyrthosiphon pisum*) | NC_012751 |
| *Pantoea ananatis* LMG 20103 | NC_013956 |
| *Pectobacterium atrosepticum* SCRI1043 | NC_004547 |
| *Photorhabdus luminescens subsp. laumondii* TTO1 | NC_005126 |
| *Proteus mirabilis* HI4320 | NC_010554 |
| *Salmonella enterica*  serovar Typhimurium LT2 | NC_003197 |
| *Serratia proteamaculans* 568 | NC_009832 |
| *Sodalis glossinidius* str. morsitans | NC_007712 |
| *Wigglesworthia glossinidia* (*Glossina brevipalpis*) | NC_004344 |
| *Xenorhabdus bovienii* SS-2004 | NC_013892 |
| *Yersinia enterocolitica* subsp. *enterocolitica* 8081 | NC_008800 |
| **Ascomycetes** |  |
| *Saccharomyces cerevisiae* | NC_001136 |
| *Schizosaccharomyces pombe* | NC_003424 |
