# Supplementary material for: Quantification of codon selection for comparative bacterial genomics
Source: BMC Genomics. 2011 Jul 25;12:374. doi: 10.1186/1471-2164-12-374 (PMC3162537; doi:10.1186/1471-2164-12-374)
Supplement: Additional file 10 — Table S5. Correlation of whole-genome measures of codon selection with tRNA count and with each other. [file 1471-2164-12-374-S10.DOC]

**Table S5**. Correlation of whole-genome measures of codon selection with tRNA count and with each other.

|  | ACE χ^2^ (Core) | ACE χ^2^  (40) | Rocha’s  ENC_diff_ | Dethlefsen’s  ΔN′_c_ | Sharp’s  S |
| --- | --- | --- | --- | --- | --- |
| Enterobacteriaceae, 14 genomes, 1060 core genes | | | |  |  |
| tRNA Count | 0.585 | 0.407 | 0.244 | 0.358 | 0.133 |
| ACE χ^2^ (Core) | 1 | 0.515 | 0.377 | 0.352 | 0.353 |
| ACE χ^2^ (40) | 0.515 | 1 | 0.005 | 0.933 | -0.069 |
| Rocha ΔENC | 0.377 | 0.005 | 1 | 0.048 | 0.609 |
| Dethlefsen’s ΔN′_c_ | 0.352 | 0.933 | 0.048 | 1 | 0.055 |
| Sharp’s S | 0.353 | -0.069 | 0.609 | 0.055 | 1 |
|  |  |  |  |  |  |
| Mycobacteriaceae, 9 genomes, 982 core genes | | | | |  |
| tRNA Count | 0.398 | 0.457 | 0.505 | 0.506 | 0.143 |
| ACE χ^2^ (Core) | 1 | 0.979 | 0.941 | 0.948 | 0.578 |
| ACE χ^2^ (40) | 0.979 | 1 | 0.972 | 0.989 | 0.525 |
| Rocha ENC_diff_ | 0.941 | 0.972 | 1 | 0.982 | 0.462 |
| Dethlefsen’s ΔN′_c_ | 0.948 | 0.989 | 0.982 | 1 | 0.438 |
| Sharp’s S | 0.578 | 0.525 | 0.462 | 0.438 | 1 |
|  |  |  |  |  |  |
| Bacilliaceae, 12 genomes, 541 core genes | | |  |  |  |
| tRNA Count | 0.561 | 0.459 | 0.337 | 0.395 | 0.421 |
| ACE χ^2^ (Core) | 1 | 0.882 | 0.887 | 0.845 | 0.878 |
| ACE χ^2^ (40) | 0.882 | 1 | 0.795 | 0.977 | 0.730 |
| Rocha ENC_diff_ | 0.887 | 0.795 | 1 | 0.761 | 0.819 |
| Dethlefsen’s ΔN′_c_ | 0.845 | 0.977 | 0.761 | 1 | 0.750 |
| Sharp’s S | 0.878 | 0.730 | 0.819 | 0.750 | 1 |
